# Practical Medicine

**Published:** 1877-04

**Authors:** 


### II. Practical Medicine.

*Paracentesis of the Pericardium, with an Analysis of Forty-one Cases.* Roberts. (*New York Med. Jour.*, December, 1876.)

Paracentesis of the pericardium, unfortunately, holds at the present time the position that was occupied by thoracentesis years ago. The operation was first proposed by Riolan, in 1649, but owing to the difficulty in making a correct diagnosis, it made but little, if any, progress until Romero's day, 1819, when it was revived. The author has collected forty-one cases which were operated on by different men, dating back to Romero in 1819, and down to Nixon in 1876. The site of operation in fifteen cases was the fifth interspace, ten the fourth, three the sixth, and two the third interspace. In the rest of the forty-one cases no seat of puncture is mentioned.

The bistoury and scissors were the first instruments used, and only three operations were made by them. The trocar came next in order, and was used seventeen times, followed by the trocar and incision six times. All these instruments of operation, however, soon gave way to the aspirator, which was first used in 1873, nine operations being made with it up to 1876. No mention is made of the instruments used on the balance of the forty-one cases reported.

Of the forty-one cases twenty recovered. Each of the fatal cases had some complication that made recovery impossible. The point of puncture should be in the fifth interspace, about midway between the left nipple and sternum, penetrating directly backward. The dangers to be most dreaded are wounding the internal mammary artery, and striking the heart as it is thrown forward by the systole; but by tapping at the point mentioned the artery is avoided, because of its situation, being about one-half or one-quarter of an inch from edge to sternum. Injury to heart could be avoided by attaching the exhausted air chamber of the aspirator to the needle as soon as it is buried beneath the skin.

The author stoutly maintains that the operation is fraught with no danger to patient, and should be made even though grave complications are present; believing that life is prolonged in the most hopeless cases, and that a cure will follow where there is no complication.

He concludes by denouncing the let-alone treatment, and says that the fluid should be removed as soon as the sac becomes filled. W. F. L.

*Scarlatina Complicated with Typhlitis*; *Fatal on the Seventh Day.* STEDMAN. (*The Boston Med. and Surg. Jour.*, December, 1876.)

Dr. B., house physician, city hospital, while attending scarlet fever cases in one of the isolated wards was stricken down with all the symptoms of scarlatina well developed.

December 5, fourth day of illness, occurred a general amelioration of the more severe symptoms, promising a speedy recovery; however, during the afternoon of same day (5th) these promises were dissipated by colicky pains around the umbilicus and in right iliac regions, making all motion of body painful.

December 7. Pain had located entirely in right iliac and hypochondriac region.

December 8. Patient's condition greatly embarrassed by hiccough, eructations and vomiting.

At 11:20 of the same day (8th) he died.

Autopsy twelve hours after death. Small quantity of purulent fluid in peritoneal cavity, also indications of recent inflammation of portion of peritoneal covering, having its origin at the appendix cœci, and slightly extending over most of right side of abdominal cavity. The appendix was adherent, much enlarged, thickened, and contained oval concretions slightly larger than a cherry stone; small intestines just above cæcum contained four or five hard yellow masses, with fæcal odor, resembling curdled milk. The kidneys were enlarged, and their cortical portion was cloudy, with slight rednesss of the cones.

Dr. S. concludes by saying that at the age of eight years the patient had scarlatina. W. F. L.

*Sulpho-Carbolate of Sodium in Diptheria.* ANTHONY. (*The Med. and Surg. Reporter*, January, 1877.)

During the past three months Dr. A. has treated eighteen cases of true diptheria successfully, save one, with sulpho-carbolate of sodium. His usual way of administering the remedy to children is to mix it with sugar and let them eat it. The dose is from one to ten grains, repeated every one, two or three hours. It may be combined with quiniæ sulph., tinct. ferri mur., ammoniæ carbon., or given in brandy, whisky, wine, syrup, or any aromatic water. W. F. L.

*Aphonia of Ten Months' Duration from Paralysis of the Arytenoideus Proprius Muscle, with Concomitant Heart Disease; Voice Restored by the Direct Application of Electricity to the Vocal Cords.* BEVERLEY. (*The American Jour. of the Med. Sciences*, Jan., 1877.)

In 1872, a lady 42 years of age, was exposed to cold, from which she lost her voice; remaining in this condition for three months, her voice spontaneously returned. Three years later (1875), her voice again disappeared, apparently from the effects of cold. Not returning this time, she consulted a physician, who pronounced it functional aphonia, and made local applications of faradic current to the throat, without effect.

May 25th, 1876, Dr. B. was called to attend patient. In connection with the faradic current to the arytenoideus muscle, which was continued every other day until June 15th, 1876, he gave strychnia 1-30 gr. three times daily. Under this treatment she improved, and in a few days was able to speak in a very good tone of voice.

Her condition rapidly improved, until she completely recovered her voice. (We are not told by the author, whether the treatment was continued with the same regularity, as from the beginning, during his attendance upon her.) At the time of first visit, patient complained of pain in left mammary region, and on pressure it was greatly augmented. Percussion and auscultation revealed some enlargement of heart, and a distinct bruit at base accompaning first sound. The author believes that, the loss of voice was due chiefly to hypertrophy of left ventricular walls; the increase of abnormal tissue, making pressure on nerve structure, resulted in reflex paralysis of one of the intrinsic muscles of larynx, effected through certain motor fibres of either vagus. W. F. L.

*Rupture of the Healthy Œsophagus.* FITZ. (*The American Jour. of The Med. Sciences*, Jan., 1877.)

A gentleman, 31 years of age, merchant, while eating, became strangulated by a tough piece of meat, about an inch in length, by one-half inch in width. After remaining in this condition about an hour, he succeeded, by great muscular energy, in ejecting it. Large clots of clotted and liquid blood were immediately expelled; this was soon followed by tumefaction of left side of neck, just below angle of lower jaw; a corresponding swelling of opposite side of neck soon followed, with prostration to patient. Liquids were swallowed without annoyance. He died on the seventh day. Autopsy showed a large longitudinal rent of œsophagus, situated in front and to right, at and below bifurcation of trachea, its length about two inches, and extending through all the coats.

W. F. L.

*Sleeping Disease.* CORRE. (*Jour. de Méd. et de Chirur. prat.*, Jan., 1877.)

Hitherto this disease has only been recognized in the negro race. The patients, at first, present a peculiar tendency to sleepiness; they become taciturn, lose appetite, and are gradually enfeebled. Finally, the tendency to sleep becomes invincible and continued. Even when awakened the affected return at once to the condition of sleep. General intelligence is fairly preserved and delirium only supervenes exceptionally. In some cases convulsions, contracture and trembling result. There is concurrent fever, especially in the evening, but without regular accesses. The issue is generally fatal, in consequence of the progressive debility. The lesions discovered are quite variable. Most frequently there is softening of the cerebral tissue, and congestion of the meninges, but as an exception, it may be noted that the cerebral substance has been found in a normal condition, with anæmic membranes. The author, calls attention to two points of etiological interest: 1. The frequency of the disease in certain definite regions, such as Western Africa, Senegambia, etc. 2. The predilection of the disease for the blacks. It has, accordingly, been considered as one of the manifestations of paludal intoxication, and it would seem to be the only manifestation of such a condition in the negroes who, as is well known, possesses a certain immunity against impaludism. Corre, however, considers this hypnosis to be due to a slow intoxication, resulting from the alimentation of the negro in those latitudes—his food consisting largely of rice, maize, etc. If this latter factor could not be demonstrated as efficient, we should perhaps refer the disease to the profoundly depressing influence of a nostalgia, much more active among the blacks than is commonly supposed to be the case. Other authors have concluded that the disease resulted from a form of encephalitis. M. Ulecia, as the result of his observations in Cuba, believes that the disease is an ischemic neurosis, and that the functional trouble is due to perverted innervation, produced by alteration of the blood.

*Inoculability of Tubercle.* METZQUER. (*Lyon Médical*, No. 23, Dec. 3, 1876.)

An interesting paper upon this subject was read by the author before the Paris Academy of Medicine. His conclusions were that tuberculosis is not a specific disease, and is not inoculable. The so-called ganulations produced by inoculation are simply infarctus, resulting from primary or secondary embolism. They are produced at the site of inoculation, either by admission to a veinule or lymphatic capillary. The solid particle, transported to the lung, may become there implanted and give origin after a fashion to a tuberculous lesion. Ordinarily, however, we find merely clots in a retrogressive phase. If the animal be permitted to survive for a time, these purely inflammatory lesions do not fail to disappear. It is only bv destroying animals at periods more or less remote from the date of the experiment, that the production of the phenomena described can be properly studied.

